# RATES OF DISENROLLMENT FROM MEDICARE MANAGED CARE PLANS ARE HIGHER AMONG RACIAL, ETHNIC, AND LINGUISTIC MINORITIES

**DOI:** 10.1093/geroni/igz038.1604

**Published:** 2019-11-08

**Authors:** Steven Martino, Megan Mathews, Cheryl Damberg, Judy Ng, Denis Agniel, Loida Tamayo, Shondelle Wilson-Frederick, Marc N Elliott

**Affiliations:** 1 RAND Corporation, Pittsburgh, Pennsylvania, United States; 2 RAND Corporation, Santa Monica, California, United States; 3 National Committee for Quality Assurance, Washington, District of Columbia, United States; 4 Centers for Medicare & Medicaid Services, Baltimore, Maryland, United States

## Abstract

Voluntary disenrollment from Medicare managed care (Medicare Advantage; MA) plans is related to beneficiaries’ negative experiences with their plan, disrupts continuity of care, and conflicts with goals to reduce Medicare costs. Information on associated factors may help illuminate the dynamics that drive decisions to disenroll. We used data from 17,517,852 beneficiaries enrolled in 736 MA plans in 2015 to investigate differences in rates of disenrollment by race, ethnicity, and preferred language. Disenrollment data came from Medicare’s enrollment system. Social Security Administration data on race and ethnicity were augmented with surname, address, and other Medicare administrative data to calculate probabilities of membership in seven race/ethnicity/language-preference groups: White, Black, English-preferring Hispanic, Spanish-preferring Hispanic, Asian or Pacific Islander (API), American Indian or Alaska Native, and multiracial. We summarized disparities across groups using regression models with and without plan intercepts, controlling for gender, disability, and Medicaid eligibility. Adjusted rates of disenrollment were significantly higher for Spanish-preferring Hispanics (19.1%), Blacks (10.2%), and APIs (9.4%) than for Whites (7.7%), and significantly lower for English-preferring Hispanics (7.4%, p’s<0.001). Within-plan disparities accounted for only a small fraction of overall disparities, indicating that Spanish-preferring Hispanics, Blacks, and APIs tended to be enrolled in plans with higher disenrollment than plans in which Whites were enrolled, whereas English-preferring Hispanics tended to be enrolled in plans with lower disenrollment. These between-plan differences may indicate that high-minority-enrollment plans less effectively inform beneficiaries about the cost and coverage of care or that racial/ethnic/linguistic minorities more often select plans that raise rates or restrict coverage.

